# Peripheral microangiopathy in precapillary pulmonary hypertension: a nailfold video capillaroscopy prospective study

**DOI:** 10.1186/s12931-021-01622-1

**Published:** 2021-01-21

**Authors:** Alexandra Arvanitaki, George Giannakoulas, Eva Triantafyllidou, Christos Feloukidis, Afroditi K. Boutou, Alexandros Garyfallos, Haralambos Karvounis, Theodoros Dimitroulas

**Affiliations:** 1 Department of Cardiology III - Adult Congenital and Valvular Heart Disease, University Hospital Muenster, Albert-Schweitzer-Campus 1, 48149 Muenster, Germany; 2 Department of Cardiology, AHEPA University Hospital, Medical School, Aristotle University of Thessaloniki, 1 St. Kyriakidi Street, 54636 Thessaloniki, Greece; 3 Fourth Department of Internal Medicine, Hippokration University Hospital, Medical School, Aristotle University of Thessaloniki, 49 Konstantinoupoleos Street, 54642 Thessaloniki, Greece; 4 Department of Respiratory Medicine, G. Papanikolaou Hospital, Thessaloniki, Greece

**Keywords:** Precapillary pulmonary hypertension, Idiopathic pulmonary hypertension, Chronic thromboembolic pulmonary hypertension, Nailfold video-capillaroscopy, Peripheral microangiopathy

## Abstract

**Background:**

Although pulmonary vascular bed has been the main subject of research for many years in pulmonary hypertension (PH), interest has recently started to divert towards the possibility of a co-existing peripheral microangiopathy. The aim of the current study was to investigate the presence of nailfold video-capillaroscopic (NVC) structural changes in patients with precapillary PH and to identify possible associations of NVC measurements with markers of disease severity.

**Methods:**

Α prospective case–control study was performed in 28 consecutive patients with precapillary PH [14 with idiopathic pulmonary arterial hypertension (IPAH) and 14 with chronic thromboembolic pulmonary hypertension (CTEPH)] and 30 healthy controls. NVC quantitative and qualitative parameters were evaluated using Optilia Digital Capillaroscope. To ensure inter-observer repeatability capillaroscopic images were reviewed by two independent investigators. For multiple comparisons among continuous variables, one-way ANOVA or the Kruskal–Wallis test were used. Differences between the groups were tested with post-hoc analysis with adjustment for multiple comparisons (Bonferroni test).

**Results:**

Both IPAH (71.4% were women, mean age 53.1 ± 13.4 years) and CTEPH (64.3% women, mean age 60.9 ± 14.4 years) groups presented reduced capillary density compared to healthy controls (8.4 ± 1.2 loops/mm and 8.0 ± 1.2 loops/mm vs. 9.7 ± 0.81 loops/mm, p < 0.001) and increased loop width (15.7 ± 3.9 μm and 15.8 ± 1.9 μm vs. 11.5 ± 2.3 μm, p < 0.001). More than half of patients with IPAH presented microhaemorrhages on capillary nailfold, while increased shape abnormalities in capillary morphology and more capillary thrombi per linear mm were detected in patients with CTEPH compared to patients with IPAH and healthy controls. All PH patients presented a non-specific NVC pattern compared to controls (p < 0.001).

**Conclusion:**

The findings of the study reveal a degree of significant peripheral microvascular alterations in patients with IPAH and CTEPH, suggesting a generalized impairment of peripheral microvasculature in pulmonary vascular disease.

## Background

Precapillary pulmonary hypertension (PH) represents a pulmonary vasculopathy defined by elevated mean pulmonary arterial pressure (mPAP) > 20 mmHg, normal pulmonary artery wedge pressure (PAWP) ≤ 15 mmHg and elevated pulmonary vascular resistance (PVR) ≥ 3 Wood Units at rest according to the 6th World Symposium on Pulmonary Hypertension Task Force [1]. Shear stress and hypoxia trigger pulmonary endothelial dysfunction and inflammation, which in turn promote the remodeling of small and medium sized pulmonary arterioles culminating in progressive obstructive pulmonary vasculopathy [2]. Chronic thromboembolic pulmonary hypertension (CTEPH), develops from the occlusion of the pulmonary vascular bed with non-resolving thromboemboli, leading also to pulmonary vascular remodeling and increased PVR [3].

Although pulmonary vascular bed has been the main subject of research for many years [4, 5], interest has recently shifted towards the possibility of a co-existing peripheral microangiopathy [6]. Nailfold video-capillaroscopy (NVC) is an established, validated, non-invasive imaging technique for the assessment of peripheral microcirculation, aiding in distinguishing different types of nailfold microvascular abnormalities [7]. It has been originally used in the assessment of Raynaud’s phenomenon and the diagnosis of systemic sclerosis (SSc) [8], in which NVC changes may serve as an early prognostic marker that determines the risk of developing pulmonary arterial hypertension (PAH) [9–11]. Moreover, a few NVC studies demonstrated evidence of a more severe peripheral microvascular dysfunction in patients with PAH associated with SSc, compared to SSc individuals without PAH, suggesting that changes in peripheral microcirculation may parallel pulmonary microangiopathy in patients with PAH [6, 12–15]. However, there are no data regarding capillary rarefaction across the various types of precapillary PH including CTEPH, whistle a handful of studies have reported on the presence of peripheral vasculopathy in patients with idiopathic PAH (IPAH) [12, 15].

The aim of the present study was to: (a) investigate the presence of peripheral microangiopathy in patients with IPAH and CTEPH compared to healthy controls, (b) explore NVC structural differences between IPAH and CTEPH, (c) identify possible associations of NVC characteristics with clinical, functional, biochemical, echocardiographic and hemodynamic parameters of disease severity in patients with precapillary PH.

## Methods

### Study design, setting, participants

This was a prospective case–control observational study which took place at a tertiary center for PH in collaboration with a tertiary center for rheumatic diseases with expertise in NVC in Northern Greece [16]. Adult patients diagnosed with IPAH or CTEPH according to the classification of 6th World Symposium on Pulmonary Hypertension Task Force [1] and healthy controls were enrolled between February 2019 and April 2020. Written informed consent was obtained from all participants. The study received approval from the Aristotle University of Thessaloniki Ethics Committee, and was performed according to the Declaration of Helsinki.

Patients with precapillary PH were either newly diagnosed and treatment naïve, as far as PAH specific therapy was concerned, or had established PH and have been receiving specific PAH medication. All participants underwent right heart catheterization—for diagnostic or follow-up purpose—at the time of study enrolment, whereas CTEPH individuals had ventilation/perfusion lung scintigraphy or computed tomographic pulmonary angiography to confirm diagnosis.

Physical examination, 12-lead electrocardiogram, six-minute walking test (6MWT), laboratory evaluation including N-terminal pro-brain natriuretic peptide, transthoracic echocardiography (TTE), spirometry with measurement of carbon dioxide diffusing capacity and NVC performed at the same 1-week time interval, during their scheduled outpatient visits. Healthy controls underwent medical history, clinical examination and NVC.

### Transthoracic echocardiography

TTE was performed on all patients to assess right heart dysfunction using Vivid S70 (General Electric, Norway) based on the recommendations for the echocardiographic assessment of the right ventricle [17]. Right ventricle (RV)-focused apical four-chamber views were obtained. RV end-diastolic area (RV EDA) and RV end-systolic area (RV ESA) were measured. The RV fractional area change (FAC) was calculated as: (RV diastolic area − RV systolic area)/RV diastolic area × 100%. Tricuspid annular plane systolic excursion [18] was acquired with M-mode placed on the lateral wall of the tricuspid annulus in the apical four-chamber view. Systolic displacement was measured from end-diastole to end-systole. In addition, tissue Doppler imaging (TDI) was applied on the lateral side of the tricuspid annulus. RV myocardial performance index (MPI) was calculated as follows: (isovolumic contraction time + isovolumic relaxation time)/RV ejection time [19].

### Nailfold videocapillaroscopy technique and image analysis

NVC was performed at room temperature (22–23 °C) with the subject seated and resting for 15 min. Subjects were asked to refrain from smoking and drinking alcohol or caffeinated drinks for at least 8 h. Optilia Digital Capillaroscope (Optilia Instruments AB, Sollentuna, Sweden) was used for image acquisition (× 200 magnification). At least two adjacent fields of 1 mm in the middle of the nailfold were captured from all hands excluding thumbs. One drop of immersion oil was applied to the nailfold to maximize the transparency of the keratin layer. In total, 16 images from each patient were subsequently captured, coded, saved and manually analyzed using Optipix Lite software (Optilia Instruments AB). To ensure inter-observer repeatability the stored pictures were reviewed by two independent investigators (ΕΤ and AΒ) blinded to the clinical data.

NVC images were quantitatively and qualitatively assessed. The following quantitative parameters were measured: capillary density (number of capillary loops per linear mm measured in the distal row following the 90° method) [20], avascular areas [21] (distinct areas in the nailfold where two or more capillaries are missing), capillary dimensions (total capillary width, arterial limb width, venous limb width, apical limb width and capillary length [21]; the mean value of each dimension of all capillaries per linear mm was eventually calculated), hemorrhages (defined as the presence of at least one hemorrhage in at least two different NVC images) and number of hemorrhages per linear mm, thromboses (number of thrombi per linear mm), edema (defined as the presence of edema in ≥ 50% of assessed fingers), capillary arrangement (capillary disorganization per linear mm defined as architectural disorientation) and capillary morphology (abnormal capillary shapes per linear mm including ramified, branched, bushy capillaries or other morphology that did not apply to normal shape). Tortuous or crossing capillaries were considered as non-specific variations of normal shapes.

As irregularly enlarged were characterized capillaries with apical limb width > 20 μm and < 50 μm and as giant, homogeneously enlarged capillaries with apical limb width ≥ 50 μm [22]. The mean of each capillaroscopic feature was calculated from the sum of consecutive images for each digit. Subsequently, the average values from eight fingers were added together and divided by the number of studied digits. The resulting value indicated the number of this capillaroscopic feature adjusted by each millimeter of the nailfold.

The “overall pattern recognition” was qualitatively assessed based on capillary density, the presence of irregularly enlarged capillaries, hemorrhages and shape abnormalities using a fast-track algorithm proposed by Smith et al. [7, 23]. Images were classified as “normal pattern”, “non-specific pattern” and “scleroderma pattern”. Furthermore, a semi-quantitative rating scale was adopted (score:0–3) based on these four capillaroscopic parameters in order to classify patients according to the severity of systemic microvascular disease. Capillary density was rated as follows: 0 for > 9 capillaries/mm, 1 for 7–9 capillaries/mm, 2 for 4–6 capillaries/mm and 3 for 1–3 capillaries per mm [24, 25]. Irregularly enlarged capillaries, hemorrhages and shape abnormalities were scored accordingly: 0 equals to no changes, 1 to changes less than 33% of the total number of capillaries/mm, 2 to changes between 33 and 66% of the total number of capillaries/mm, and 3 to changes more than 66% of the total number of capillaries/mm [24, 25]. Total score was calculated by the sum of scores for each finger divided by the total number of fingers evaluated and was rounded to the next integer to define the risk group.

### Statistical methods

Data are presented as mean ± standard deviation or as median (interquartile range, IQR) for continuous variables. Normal distribution was assessed using the Shapiro–Wilk test. Differences between two independent categories with respect to quantitative variables were analysed using the Student’s *t* test for independent variables or the Mann Whitney test. For multiple comparisons between three independent groups, one-way ANOVA or the Kruskal–Wallis test were used. Differences between the groups were tested with post-hoc analysis with adjustment for multiple comparisons (Bonferroni test). The reproducibility of quantitative capillaroscopic characteristics (capillary density, capillary loop diameter and capillary shape abnormalities) was tested by measuring agreement between the two independent investigators using Bland–Altmann analysis, which did not reveal significant inter-observer variability (Additional file 1).

Categorical variables are presented as absolute count and percentage (%) and were analysed using the chi-square test or Fisher’s exact test when appropriate. The reproducibility of the semi-quantitative rating scale was evaluated using Cohen's kappa coefficient, with a value of 0.82 indicating a good inter-observer agreement in the overall evaluation of capillaroscopic findings.

Pearson or Spearman coefficient (r) was used to explore the correlation between capillaroscopic parameters and functional, laboratory, echocardiographic and hemodynamic parameters. For normally distributed variables linear equation with 95% confidence intervals (CI) were also presented. A p-value < 0.05 was considered statistically significant. Data were analysed using IBM SPSS statistics (version 26.0) software.

## Results

### Baseline characteristics

In total, 28 consecutive patients with precapillary PH (14 patients with IPAH and 14 patients with CTEPH, 67.8% women, mean age 56.6 ± 14.1 years) and 30 healthy controls were included in the study (Table 1). About half of PH patients [46.4%, (13/28)] were in World Health Organization Functional Class (WHO) II. Mean oxygen saturation at rest (SpO2%) was 93.2 ± 4.1%, while diffusion lung capacity for carbon monoxide (DLCO) was also reduced (65.9 ± 20.2%). Hemodynamics did not significantly vary between IPAH and CTEPH patients. Median NT-proBNP was increased in patients with CTEPH compared to those with IPAH.

**Table 1:** Baseline characteristics of patients with precapillary PH and controls

Variables	Total PH cohort	IPAH	CTEPH	Healthy controls	P-value_1_*	P-value_2_**
N	28	14	14	30		
Female, n (%)	19 (67.8)	10 (71.4)	9 (64.3)	21 (70.0)	0.87	0.75
Age, y	56.6 ± 14.1	53.1 ± 13.4	60.9 ± 14.4	50.6 ± 13.4	0.45	0.08
BMI, kg/m^2^	29.8 ± 5.4	28.8 ± 6.8^$^	30.4 ± 3.8^#^	24.7 ± 3.1^$#^	< 0.001	< 0.001
SpO_2_% rest	93.2 ± 4.1	93.7 ± 4.9	92.6 ± 3.2			0.58
WHO FC, n (%)						
I	3 (10.7)	2 (14.2)	1 (7.1)			0.61
II	13 (46.4)	6 (42.8)	7 (50.0)			
III	11 (39.3)	5 (35.7)	6 (42.9)			
IV	1 (3.5)	1 (7.1)	0			
6-MWD, (m)	429.8 ± 125.3	471.3 ± 133.1	382.5 ± 103.8			0.14
eGFR, ml/min/1.73 m^2^	81.2 (18.3)	78.6 (17.6)	87.9 (18.8)			0.34
NT-proBNP (pg/ml)	219 (393)	188 (2005)	335 (361)			< 0.001
Heamodynamics						
mRAP, mmHg	7.7 ± 4.3	6.9 ± 4.1	8.5 ± 4.6			0.39
mPAP, mmHg	44.3 ± 13.5	44.4 ± 16.3	44.1 ± 10.7			0.97
PAWP, mmHg	11.1 ± 2.7	10.6 ± 1.9	11.7 ± 2.2			0.34
CI, ml/m^2^	3.0 ± 0.7	3.2 ± 0.7	2.7 ± 0.6			0.08
PVR, WU	5.7 (5.3)	5.8 (5.7)	5.7 (4.8)			0.64
Echocardiography						
RV FAC%	30.5 ± 9.3	26.8 ± 9.7	33.1 ± 7.9			0.07
TAPSE, mm	18.9 ± 6.2	19.1 ± 7.5	18.8 ± 4.9			0.89
RV MPI	0.37 (0.15)	0.39 (0.2)	0.36 (0.12)			0.45
Lung function test						
FEV1/FVC %	82.7 ± 8.2	84.1 ± 9.7	83.6 ± 7.7			0.48
DLCO %	65.9 ± 20.2	66.4 ± 25.2	64.9 ± 6.4			0.92
PAH treatment	17 (60.7)	11 (78.6)	6 (42.8)			0.048
Monotherapy	10 (35.7)	5 (41.6)	5 (35.7)			
Dual therapy	4 (14.3)	4 (28.5)	0			
Triple therapy	3 (10.7)	2 (14.3)	1 (7.1)			

Categorical variables are presented as frequency and percentage, n (%)

Continuous variables are presented as mean value ± standard deviation or median value with interquartile range (IQR)

*BMI* body mass index, *6-MWD* 6-min walk distance, *bpm* beats per minute, *CI* cardiac index, *CTEPH* chronic thromboembolic pulmonary hypertension, *DLCO* diffusing capacity for carbon monoxide, *FAC* fractional area change, *FC* functional class, *FEV1* forced expiratory volume during the first second of expiration, *FVC* forced vital capacity, *GFR* glomerular filtration rate, *IPAH* idiopathic pulmonary arterial hypertension, *mPAP* mean pulmonary artery pressure, *MPI* myocardial performance index, *mRAP* mean right atrial pressure, *NT-proBNP* N-terminal pro-brain natriuretic peptide, *PAH* pulmonary arterial hypertension, *PAWP* pulmonary artery wedge pressure, *PVR* pulmonary vascular resistance, *RV* right ventricle, *SpO*_*2*_*%* arterial oxygen saturation, *TAPSE* tricuspid annular plane systolic excursion, *WHO* World Health Organization, *WU* wood units

*P-value_1_ refers to difference between total pre-capillary PH cohort and healthy controls

**P-value_2_ refers to difference among IPAH, CTEPH and healthy controls. For variables in which there is no value in the column of healthy controls, then p-value_2_ refers to difference between IPAH and CTEPH. Statistical significance is defined as P < 0.05

About six out of ten patients [60.7%, (17/28)] received specific PAH treatment at the time of NVC examination, with more than one third being under monotherapy [35.7% (10/28)]. With regards to patients with prevalent disease, median duration from diagnosis was 11 (IQR 57) months. In CTEPH subgroup, three patients had persistent CTEPH pulmonary endarterectomy and one patient underwent multiple sessions of balloon pulmonary angioplasty.

### Capillaroscopic alterations in IPAH and CTEPH patients

**Fig. 1:**
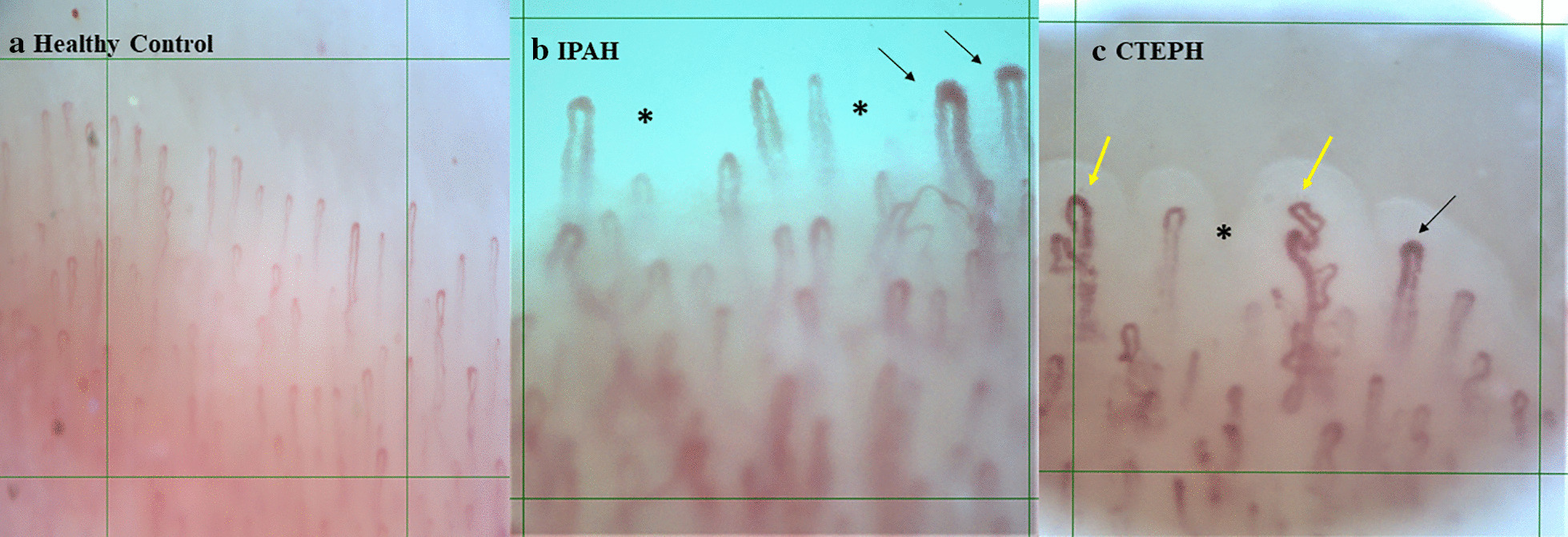
Nailfold video-capillaroscopic features in our study population. **a** Healthy control; normal capillary density (10 loops/mm), hairpin-shaped capillaries with normal loop width. **b** Idiopathic pulmonary arterial hypertension (IPAH); moderately reduced capillary density (5 capillary loops/mm), avascular areas (asterisk) and irregularly enlarged capillaries (> 20 μm and < 50 μm, black arrows). **c** Persistent chronic thromboembolic pulmonary hypertension (CTEPH) post pulmonary endarterectomy; mildly reduced capillary density (7 capillary loops/mm), avascular area (asterisk), abnormal capillary shapes (yellow arrows) and irregularly enlarged capillaries (> 20 μm and < 50 μm, black arrow). Grid pattern represents area of 1mm^2^. Magnification × 200

**Fig. 2:**
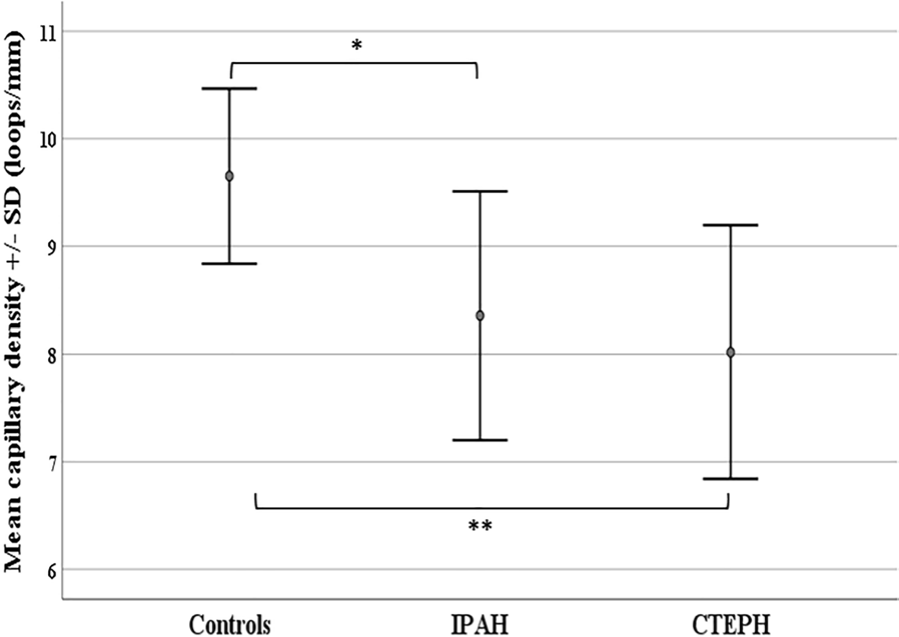
Capillary density. Error bars represent the mean capillary density ± standard deviation (SD) in healthy controls (9.7 ± 0.81 loops/mm), patients with idiopathic pulmonary arterial hypertension (IPAH) (8.4 ± 1.2 loops/mm) and patients with chronic thromboembolic pulmonary hypertension (CTEPH) (8.0 ± 1.2 loops/mm). Difference in capillary density was significant among the three groups (p < 0.001). In detail, patients with IPAH presented significantly lower capillary density compared to healthy controls (*p < 0.01). In addition, patients with CTEPH presented significantly lower capillary density compared to healthy controls (**p < 0.001). No difference in capillary density was found between IPAH and CTEPH patients

**Fig. 3:**
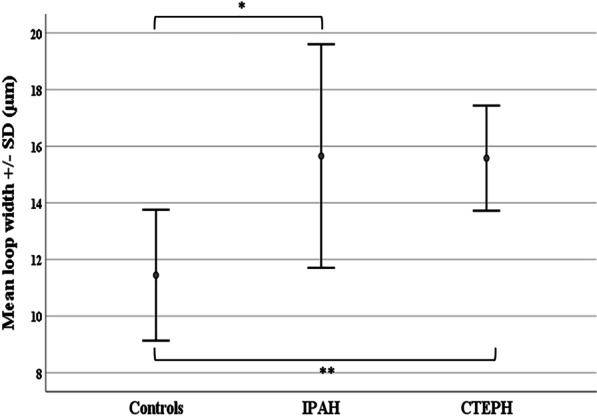
Loop width. Error bars represent the mean loop width ± standard deviation (SD) in healthy controls (11.5 ± 2.3 μm), patients with idiopathic pulmonary arterial hypertension (IPAH) (15.7 ± 3.9 μm) and patients with chronic thromboembolic pulmonary hypertension (CTEPH) (15.8 ± 1.9 μm). Difference in loop width was significant among the three groups (p < 0.001). In detail, patients with IPAH presented significantly increased loop width compared to healthy controls (*p < 0.001). In addition, patients with CTEPH presented increased loop width compared to healthy controls (**p < 0.001)

The majority of capillaroscopic parameters were abnormal in patients with IPAH and CTEPH as compared to healthy controls (Fig. 1, Table 2). Both groups of patients presented significantly reduced capillary density (Fig. 2) and increased capillary dimensions, namely capillary width, loop width (Fig. 3), arterial and venous limb width compared to controls. Avascular areas were also observed in the majority of patients with IPAH and CTEPH. Irregularly enlarged capillaries were also present in a significant number per linear mm in both groups of patients compared to controls, while no giant capillaries were observed in the study cohort. Capillary length was found to be in normal range.

**Table 2:** Capillaroscopic abnormalities in patients with idiopathic pulmonary arterial hypertension and chronic thromboembolic pulmonary hypertension

	IPAH	CTEPH	Healthy controls	P-value*
N	14	14	30	
Avascular areas (n/mm)	0.75 ± 0.26^+^	0.67 ± 0.42^#^	0.15 ± 0.2^+^^#^	< 0.001
Avascular areas, n (%)	13 (92.8)	12 (85.7)	8 (26.6)	< 0.001
Capillary width (μm)	35.1 ± 5.1^+^	39.2 ± 4.6^#^	29.5 ± 4.3^+^^#^	< 0.001
Arterial limb (μm)	10.2 ± 2.2^+^	10.3 ± 1.5^#^	7.4 ± 1.3^+^^#^	< 0.001
Venous limb (μm)	12.4 ± 2.4^+^	12.6 ± 2.4^#^	8.8 ± 1.4^+^^#^	< 0.001
Irregularly enlarged (loops/mm)	1.4 ± 1.2^+^	1.2 ± 0.7^#^	0.4 ± 0.5^+^^#^	< 0.001
Capillary length (μm)	323.8 ± 84.9	315.2 ± 107.7	313.2 ± 83.9	0.99
Edema, n (%)	3 (21.4)	0	0	–
Microhemorrhages (n/mm)	0.25 (0.25)	0.12 (0.4)	0 (0)	< 0.001
Microhemorrhages, n (%)	8 (57.1)	5 (35.7)	0	< 0.001
Thrombosis (n/mm)	2.2 ± 0.8^$^	3.2 ± 1.3^$#^	1.4 ± 0.8^#^	< 0.001
Disorganized capillaries (loops/mm)	0.5 ± 0.4	0.4 ± 0.3	0.2 ± 0.4	0.079
Shape abnormalities (loops/mm)	1.8 ± 1.0^$^	3.1 ± 1.2^$#^	1.1 ± 0.8^#^	< 0.001
Ramified capillaries (loops/mm)	0.5 ± 0.4^+^	0.7 ± 0.3^#^	0.2 ± 0.2^+^^#^	< 0.001

Categorical variables are presented as frequency and percentage, n (%)

Continuous variables are presented as mean value ± standard deviation or median value and interquartile range

*IPAH* idiopathic pulmonary arterial hypertension, *CTEPH* chronic thromboembolic pulmonary hypertension

*A p-value < 0.05 is considered statistically significant. Comparisons were made among the three groups

^+^Statistical significance between IPAH and controls. P-value < 0.01

^#^Statistical significance between CTEPH and controls. P-value < 0.01

^$^Statistical significance between CTEPH and IPAH. P-value < 0.01

As far as qualitative characteristics were concerned, more than half of patients with IPAH and more than one third of patients with CTEPH presented microhaemorrhages on capillary nailfold, while 3 patients with IPAH had significant capillary edema (21.4%). In addition, increased abnormalities in capillary shape morphology and significantly more capillary thrombi per linear mm were detected in patients with CTEPH compared to patients with IPAH and healthy controls (Table 2). On the other hand, the number of disorganized capillaries per linear mm was not significantly increased between patients and controls.

All PH patients presented a non-specific NVC pattern compared to controls (50% of those presented a normal pattern, p < 0.001). No patient or control had a scleroderma pattern. Moreover, according to the semi-quantitative classification, the majority of patients with IPAH (71.2%) and almost all patients with CTEPH (92.8%) presented mild capillaroscopic changes (score = 1) compared to two thirds of controls that presented normal capillaries (score = 0). Finally, two patients with IPAH and one patient with CTEPH had more severe changes on capillary nailfold (score = 2), (Table 3).

**Table 3:** Semi-quantitative nailfold video-capillaroscopy severity score to evaluate capillaroscopic abnormalities in patients with IPAH, CTEPH and healthy controls

	Healthy controls (N = 30)	IPAH (N = 14)	CTEPH (N = 14)
Semi-quantitative severity score			
0	20 (66.7%)	2 (14.3%)	0
1	10 (33.3%)	10 (71.4%)	13 (92.8%)
2	0	2 (14.3%)	1 (7.2%)

A scoring system was adopted using capillary density, the presence of irregularly enlarged capillaries, hemorrhages and shape abnormalities. Total score was calculated by the sum of scores for each finger divided by the total number of fingers evaluated and was rounded to the next integer to define the risk group. Chi-square test was performed to evaluate statistical significance among the three groups. A p-value equal to 0.001 < 0.05 shows significant difference among groups

*IPAH* idiopathic pulmonary arterial hypertension, *CTEPH* chronic thromboembolic pulmonary hypertension

### Correlations between capillaroscopic characteristics and markers of cardiac function

No biochemical, functional, echocardiographic or hemodynamic variables were correlated with capillary density or other capillary features in the IPAH group.

In CTEPH, a negative linear correlation between SpO_2_% and capillary density was detected [r = − 0.58, B =− 0.2, 95% CI (− 0.4, − 0.02) p = 0.037]. In addition, RV MPI was negatively associated both with capillary density (r = − 0.69, p = 0.02) and with abnormal capillaries (r = − 0.68, p = 0.02) (Additional file 1).

## Discussion

The main finding of our study is the demonstration of peripheral microvascular impairment assessed by NVC in patients with IPAH and CTEPH. In particular, both groups of patients presented reduced capillary density and increased capillary dimensions compared to controls, with non-specific morphological markers of microvascular dysregulation of mild severity being present in the majority of PH population, indicating a generalized microangiopathy.

Certain pathogenetic mechanisms could explain the presence of systemic vasculopathy in our study cohort. For example, the imbalance between vasodilation and vasoconstrictor mediators, culminating in excessive vasoconstriction, endothelial and smooth muscle proliferation may account for pulmonary vascular remodeling as well as peripheral vascular changes in ΙPΑH [4]. Vascular endothelial growth factor (VEGF) and proinflammatory cytokines are likely mediators of chronic hypoxia-driven pulmonary and peripheral vascular remodeling in PAH, by mobilizing endothelial progenitor cells (EPCs) [26–29]. A severe reduction in circulating EPCs despite VEGF stimulus in late stages of SSc and its positive correlation with the reduction in capillary density may explain peripheral microvascular alterations [28], observed among entities of precapillary PH in our study.

Limited amount of data point towards the presence of a widespread vascular injury as determined by forearm blood flow dilation after brachial artery occlusion in PAH [30, 31]. The majority of existing NVC studies until today have focused on systemic microvascular changes in PAH associated with SSc, by demonstrating significant reduction in capillary density accompanied by an increase in capillary loop width, both of which have been correlated with indices of hemodynamic severity such as mPAP [12, 15, 32]. In the context of IPAH, Hofstee et al. reported a significant decrease in capillary density between 20 patients with IPAH and 21 healthy controls [15], and these observations were confirmed by Corrado et al., who also displayed increased loop width in IPAH patients [12].

The present study supports and further expands previous observations by establishing reduced capillary density and increased loop width as a common NVC feature amongst patients with IPAH and CTEPH. Moreover, other morphological markers of microvascular dysregulation, namely irregularly enlarged capillaries and microhaemorrhages were detected among IPAH and CTEPH patients at a greater extent compared to controls, whereas capillary edema has been identified for the first time in some patients with IPAH. Taking into consideration that cardiac index was preserved and mean right atrial pressure was below 8 mmHg in our PH cohort, the abnormal capillaroscopic patterns are rather unlikely to be explained on the basis of decreased right ventricular cardiac output and could be attributed to a generalised vasculopathy. Further large longitudinal studies would determine whether NVC changes represent a parameter of global microvascular damage or reflect low blood flow due to impaired cardiac performance in PH patients.

Besides obstructive macrovascular disorder, CTEPH also encapsulates a “secondary arteriopathy” component of small sized pulmonary arterioles in which inflammation, oxidative stress and endothelial dysfunction play an important role [29]. In the current study, patients with CTEPH presented not only with mild capillaroscopic alterations similar to NVC changes observed in IPAH, but also with a higher degree of abnormal capillaries and capillary thrombi compared to IPAH patients. Taking into account the negative linear correlation between rest arterial oxygen saturation and capillary density, enhanced NVC abnormalities in CTEPH group could be explained on the basis of a possible hypoxia-induced peripheral vasculogenesis [33, 34]. On the other hand, the negative correlation of RV MPI with capillary density and abnormal capillary shapes may indicate a possible association of impaired right ventricular function and diminished vascularity. Taking altogether, these novel findings suggest the presence of an impaired peripheral microcirculation in CTEPH and may shed new light on the pathophysiology of the disease.

The main limitation of our study is the small sample size, which could explain the lack of a significant association between capillaroscopic and functional or hemodynamic markers of cardiac dysfunction. The structured study protocol has considerably contributed to the low number of participants. In this way we secured that haemodynamic and capillaroscopic data were recorded at the same time interval, which is very important for the reliability of the results. Another limitation is that the majority of patients have been receiving PAH specific therapy, which could be a confounding factor for NVC changes. As a result, we could not signify if the presence of certain non-specific morphological abnormalities observed in PH patients, such as microhaemorrhages, thrombi and shape abnormalities may indicate disease progression or treatment response.

A strength of our study is that NVC was conducted in all fingers except thumbs and the acquisition of two adjacent images from each finger according to the updated European League against Rheumatism (EULAR) recommendations [7]. Several studies acquired images only from the fourth finger of the non-dominant hand, which limited the generalizability of their results [15, 35]. In addition, qualitative and semi-quantitative assessment was performed based on a validated algorithm proposed by Smith et al., which facilitates parameters that can be reliably measured by a trained examiner [7, 23, 36]. Furthermore, two independent investigators conducted the image analysis with a good inter-observer agreement. Last but not least, this is one of only few studies that comprehensively analyzed in a systemic manner all capillaroscopic features in both IPAH and CTEPH patients.

## Conclusion

This study demonstrated significant NVC microvascular changes in patients with IPAH and CTEPH suggestive of an impaired peripheral microcirculation. Further prospective multi-center studies are warranted to confirm our results and reveal potential NVC markers as predictors of clinical outcomes in PH cohorts.

## Supplementary Information

**Additional file 1: Figure 1.** Bland–Altmann analysis was performed to identify inter-observer variability in capillary density (loops/mm) measurements in the study cohort. **Figure 2.** Bland–Altmann analysis was used to identify inter-observer variability in loop diameter (μm) measurements in the study cohort. **Figure 3.** Bland–Altmann analysis was performed to identify inter-observer variability in measuring the number of shape abnormalities per linear mm in the study cohort. **Table.** Correlations among capillaroscopic parameters and demographic, laboratory, functional, echocardiographic and heamodynamic markers of cardiac function in patients with precapillary PH.

## Data Availability

The datasets used and/or analysed during the current study are available from the corresponding author on reasonable request.

## Abbreviations

CTEPH: Chronic thromboembolic pulmonary hypertension
EPCs: Endothelial progenitor cells
EULAR: European League against Rheumatism
ESC/ERS: European Society of Cardiology/European Respiratory Society
FAC: Fractional area change
IPAH: Idiopathic pulmonary arterial hypertension
IQR: Interquartile range
6-MWT: Six-minute walking test
mPAP: Mean pulmonary arterial pressure
MPI: Myocardial performance index
NVC: Nailfold video-capillaroscopy
PAWP: Pulmonary artery wedge pressure
PH: Pulmonary hypertension
PVR: Pulmonary vascular resistance
RV: Right ventricle
RV EDA: Right ventricular end-diastolic area
RV ESA: Right ventricular end-systolic area
SSc: Systemic sclerosis
SpO2%: Arterial oxygen saturation
TAPSE: Tricuspid annular plane systolic excursion
TDI: Tissue Doppler imaging
VEGF: Vascular endothelial growth factor
