# Supplementary material for: Peripheral microangiopathy in precapillary pulmonary hypertension: a nailfold video capillaroscopy prospective study
Source: Respir Res. 2021 Jan 21;22:27. doi: 10.1186/s12931-021-01622-1 (PMC7819216; doi:10.1186/s12931-021-01622-1)
Supplement: Supplementary file 1 — Additional file 1: Figure 1. Bland–Altmann analysis was performed to identify inter-observer variability in capillary density (loops/mm) measurements in the study cohort. Figure 2. Bland–Altmann analysis was used to identify inter-observer variability in loop diameter (μm) measurements in the study cohort. Figure 3. Bland–Altmann analysis was performed to identify inter-observer variability in measuring the number of shape abnormalities per linear mm in the study cohort. Table. Correlations among capillaroscopic parameters and demographic, laboratory, functional, echocardiographic and heamodynamic markers of cardiac function in patients with precapillary PH. [file 12931_2021_1622_MOESM1_ESM.docx]

**Additional File 1**

**Figure 1.** Bland-Altmann analysis was performed to identify inter-observer variability in capillary density (loops/mm) measurements in the study cohort.


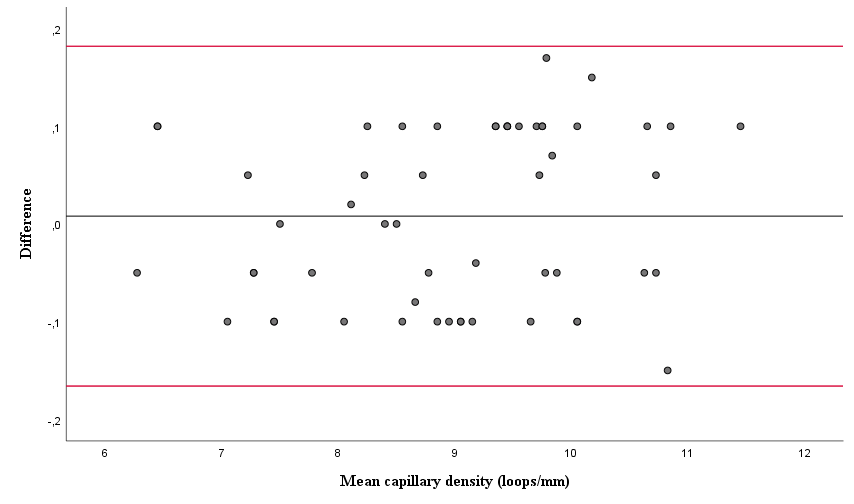


Reference line represents the mean difference in measurements between investigator 1 and 2, while the upper and lower red lines represent the 95% confidence intervals (CIs) (mean difference: 0.008 loops/mean; standard deviation: 0.09; 95% CIs: -0.16,0.18; p= 0.51).

**Figure 2.** Bland-Altmann analysis was used to identify inter-observer variability in loop diameter (μm) measurements in the study cohort.


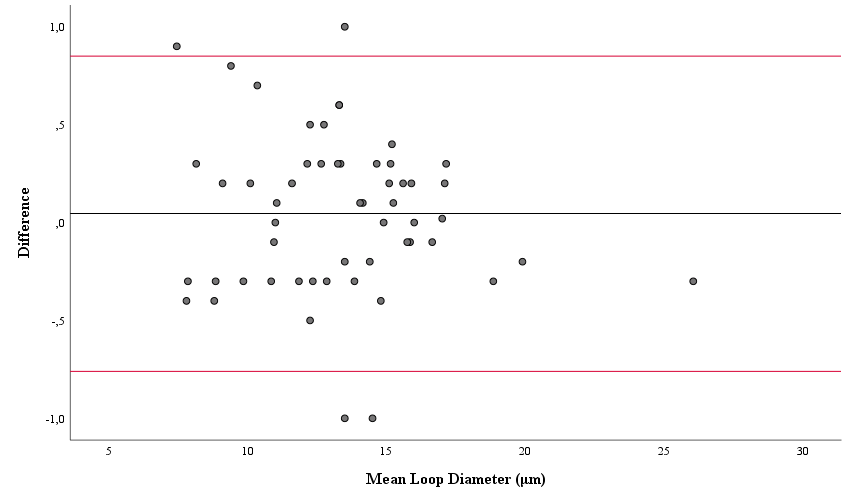


Reference line represents the mean difference in measurements between investigator 1 and 2, while the upper and lower red lines represent the 95% confidence intervals (CIs) (mean difference: 0.046μm; standard deviation: 0.41; 95% CIs: -0.76,0.85; p= 0.41).

**Figure 3.** Bland-Altmann analysis was performed to identify inter-observer variability in measuring the number of shape abnormalities per linear mm in the study cohort.


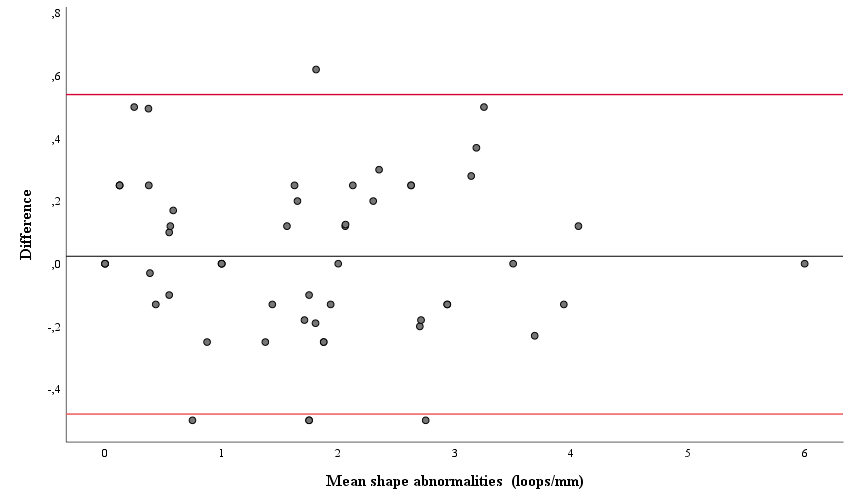


Reference line represents the mean difference in measurements between investigator 1 and 2, while the upper and lower red lines represent the 95% confidence intervals (CIs) (mean difference: 0.024 loops/mean; standard deviation: 0.26; 95% CIs: -0.48,0.54; p= 0.48).

**Table.** Correlations among capillaroscopic parameters and demographic, laboratory, functional, echocardiographic and heamodynamic markers of cardiac function in patients with precapillary PH.

|  | **IPAH** | | **CTEPH** | | | |
| --- | --- | --- | --- | --- | --- | --- |
|  | **Capillary Density (loops/mm)** | | **Capillary Density (loops/mm)** | | **Shape Abnormalities (loops/mm)** | |
|  | **Coefficient (r)** | **P-Value*** | **Coefficient (r)** | **P-Value*** | **Coefficient (r)** | **P-Value*** |
| Age, years | 0.4 | 0.19 | 0.36 | 0.19 | -0.07 | 0.81 |
| BMI, kg/m^2^ | -0.35 | 0.26 | -0.03 | 0.9 | -0.05 | 0.84 |
| SpO2% | 0.05 | 0.87 | **-0.58** | **0.04** | 0.02 | 0.94 |
| 6-MWD, m | 0.18 | 0.60 | -0.38 | 0.24 | -0.003 | 0.99 |
| Log_10_ (NT-proBNP) | -0.26 | 0.4 | 0.15 | 0.62 | -0.06 | 0.86 |
| GFR, ml/min | -0.37 | 0.22 | -0.07 | 0.78 | 0.07 | 0.81 |
| mPAP, mmHg | -0.45 | 0.13 | 0.33 | 0.24 | 0.05 | 0.86 |
| CI, L/min/m^2^ | -0.12 | 0.7 | 0.05 | 0.86 | 0.05 | 0.85 |
| PVR, WU | -0.18 | 0.55 | 0.35 | 0.24 | 0.05 | 0.87 |
| TAPSE, mm | 0.15 | 0.62 | 0.36 | 0.2 | 0.38 | 0.17 |
| RV MPI | -0.33 | 0.34 | **-0.69** | **0.02** | **-0.68** | **0.02** |

PH: pulmonary hypertension; IPAH: idiopathic pulmonary arterial hypertension; CTEPH: chronic thromboembolic pulmonary hypertension; BMI: body mass index; 6-MWD: 6-minute walk distance; bpm: beats per minute; GFR: glomerular filtration rate; Log_10_ (NT-proBNP): common logarithm of N-terminal pro-brain natriuretic peptide; MPI: myocardial performance index; RV: right ventricle; SpO2% arterial oxygen saturation %, TAPSE: tricuspid annular plane systolic excursion, mPAP: mean pulmonary arterial pressure, CI: cardiac index, PVR: pulmonary vascular resistance

*A p-value <0.05 (bold) is considered statistically significant.
